# A novel imaging-based approach for large extracellular vesicle detection and prognostic stratification in metastatic breast cancer

**DOI:** 10.1016/j.jlb.2026.100485

**Published:** 2026-07-30

**Authors:** Eszter Papp, Vincent Liégeois, Jo Vandesompele, Peter Vermeulen, Pieter Mestdagh, Mark Kockx

**Affiliations:** a CellCarta NV, Antwerp, Belgium; b Translational Cancer Research Unit, Department of Cancer Research, Oncology Centre Antwerp, ZAS Augustinus, Antwerp, Belgium; c Department of Biomolecular Medicine, Ghent University, Ghent, Belgium

**Keywords:** Liquid biopsy, Large extracellular vesicles, LEVs, Circulating tumor cells, Metastatic breast cancer, Tumor-derived extracellular vesicles, Prognostic biomarker

## Abstract

**Background:**

Large extracellular vesicles (LEVs) are membrane-bound extracellular particles released by tumour cells into body fluids. Circulating LEVs carry tumour-associated biomaterials and are more abundant than circulating tumour cells (CTCs), representing a valuable liquid biopsy analyte. We evaluated the RareCyte® CTC platform's ability to identify LEVs in blood smears and explored associations with clinicopathological features in a metastatic breast cancer (MBC) cohort.

**Methods:**

MBC patient samples (N = 72) from a previously published prospective comparison study were retrospectively re-analysed to develop and validate an LEV identification and enumeration workflow. Correlations with CellSearch® tumour-derived extracellular vesicle (tdEV) counts and CTC counts were assessed using Spearman's rho. Between-platform comparisons were performed using Wilcoxon's signed-rank test Associations with overall survival (OS) were assessed using Kaplan–Meier analysis and Cox proportional hazards models. Overall survival was defined as the interval between blood collection and death from any cause.

**Results:**

Median LEV count [IQR] was 89 [53–208], with detectable LEVs in patients with low or absent CTC counts. LEV counts positively correlated with matched CTC counts (Spearman's ρ = 0.88, p < 0.001) and CellSearch tdEV counts (Spearman's ρ = 0.80, p < 0.001). Higher LEV counts were associated with significantly shorter overall survival (p = 0.04). RareCyte LEV counts were significantly higher than CellSearch tdEV counts, potentially reflecting methodological differences between enrichment workflows. In Cox analysis, higher LEV counts were associated with shorter OS (HR per 100 LEVs = 1.04, p = 0.0015), and CTCs were also significantly associated with OS.

**Conclusion:**

We developed an operational workflow for LEV detection and quantification using the RareCyte® CTC analysis platform. The inclusion of LEVs into CTC-based liquid biopsy analyses may provide complementary biomarker information, particularly in patients with low CTC burden.

## Highlights

• The RareCyte platform can reliably detect large extracellular vesicles (LEVs).
• In MBC patients, LEV count correlated with CTCs but were significantly more abundant.
• LEV counts correlated with overall survival, indicating value in tumor monitoring.
• RareCyte-based LEVs may enable quantitative biomarker detection in liquid biopsy.

## Abbreviations:

ACCEPT -: Automated CTC Classification, Enumeration, and Phenotyping
CI: confidence interval
CK -: cytokeratin
CTC -: circulating tumor cell
DAPI -: (4′,6-diamidino-2-phenylindole) DNA stain
EpCAM -: epithelial cell adhesion molecule
EV -: extracellular vesicle
HER2: human epidermal growth factor receptor 2
HR -: hazard ratio
LEV -: large extracellular vesicle
MBC -: metastatic breast cancer
tdEV -: tumor derived extracellular vesicle

## 1 Introduction

Large extracellular vesicles (LEVs) are a distinct subset of extracellular vesicles associated with tumour cells and first described in prostate cancer [1]. These vesicles are generated through membrane blebbing, predominantly in migratory cancer cells [2], [3], [4]. LEVs are typically 1-10 μm membrane-bound vesicles containing fragmented DNA, diverse RNA species, proteins, and lipids [5], [6], and play a role in intercellular communication by transferring oncogenic material to recipient cells [4], [7], [8].

Circulating LEVs have been reported in other tumour types, including lung cancer, breast cancer, and glioblastoma [2], [3], [7], [9], [10], [11].

The CellSearch platform, originally developed for EpCAM-positive CTC detection, can identify LEV-like structures through image analysis with the automated CTC Classification, Enumeration, and Phenotyping (ACCEPT) algorithm, where they are categorized as tumor derived extracellular vesicles (tdEVs); defined as nucleus-free objects that are 1-14 μm in diameter with an eccentricity index of 0.8 or higher. The object population identified with this method is enriched for CK^+^/CD45^-^/DAPI^−^ objects within the LEV size range, although it cannot be excluded that apoptotic bodies and other objects are occasionally included. These operationally defined “tdEVs” have shown independent and complementary prognostic value across several malignancies. In breast cancer, tumor-derived EVs (tdEVs) count was reported to be significantly associated with clinical outcomes [12], [13]. A recent publication proposed the analysis of tumor burden in metastatic breast cancer and castration resistant prostate cancer combining tumor signal from CTCs and tdEVs into a single metric, blood tumor load (BTL). BTL was significantly associated with overall survival and supported the assessment of HER2 status.

An independent method, the High-Definition Single Cell Assay (HDSCA) workflow demonstrated high diagnostic and staging accuracy of large EVs in combination with CTCs, underscoring their potential as liquid biopsy analytes [3].

Despite growing interest in large extracellular vesicles as liquid biopsy analytes, standardized workflows for their detection and enumeration remain limited.

Integration of tdEVs with CTCs and circulating DNA can enhance patient stratification [9], [10], [14], [15] while biomarker characterisation revealed concordance with both CTCs and tumour tissue [6], [12].

The CellSearch system enriches blood samples for tumor-derived objects by EpCAM-based capture. EpCAM positive tumor cells or vesicles account for only a portion of the total tumor signal, especially in advanced/metastatic disease [16], [17], and are absent in several tumor types.

The AccuCyte-CyteFinder workflow developed for the RareCyte platform is an alternative high-resolution imaging system designed for CTC and rare-cell detection from fluids [18], [19]. Although optimised for CK- or EpCAM-expressing cells, its density-based enrichment and imaging workflow allow detection of other tumour-derived objects with cell-like density. The label-free capture enables the use of this platform in settings where EpCAM expression is not present.

The use of this platform to capture large extracellular vesicles would expand its use to conditions currently not analyzable with the CellSearch/ACCEPT tdEV workflow, adding a valuable tool to monitoring tumor burden in challenging diseases such as melanoma, small cell lung cancer or malignancies of the central nervous system.

As LEVs are in the size range detectable by the imaging methods utilized with the system, have a density close to that of the CTCs [20], and epithelial marker positivity, we hypothesized that these vesicles are captured by the AccuCyte-CyteFinder workflow. We speculated that the CK/EpCAM dual labelling will result in a more robust signal when detecting smaller objects, like LEVs. In addition, the manual review of the objects pre-identified by the CyteMapper algorithm enables the exclusion of apoptotic bodies and other artifacts, potentially leading to a cleaner, more reliable dataset.

As RareCyte has a broad selection of off-the-shelf biomarkers and offers a developer kit with the opportunity to include two custom biomarkers, the RareCyte platform supports the analysis of circulating tumor-derived objects with higher sensitivity, less selection bias and more detailed phenotypic characterisation compared to CellSearch.

In the present study, we evaluate the ability of the RareCyte platform to detect LEVs and compare LEV counts with CellSearch-derived tdEV counts in matched samples from patients with metastatic breast cancer (MBC). We explore the association between LEV abundance and overall survival, tumour biomarker status, and metastatic burden, providing insight into the potential of LEV in disease monitoring using a commercially available CTC analysis platform.

## 2 Methods

### 2.1 Patient samples

#### 2.1.1 Patients

This study is a secondary analysis of samples and data from a previously approved prospective study [21]. Briefly, patients with metastatic breast cancer starting a new line of treatment were enrolled at the University Hospital St. Augustinus in Antwerp, Belgium. Patient recruitment started in February 2019 and ended in December 2020. Blood samples were collected at the Translational Cancer Research Unit °(TCRU) and coded. The original study was approved by the ethical review boards of GZA Hospitals Sint-Augustinus and the University of Antwerp (file number UA A11-18), and all patients provided written informed consent for biomarker analyses and clinical data collection.

#### 2.1.2 Samples and data

Patients each donated paired samples of each 10 mL whole blood, collected in CellSave tubes or in AccuCyte tubes for analysis by CellSearch or AccuCyte-CyteFinder systems, respectively. Both tests were performed according to standardized procedures. Blood samples were stored at room temperature and processed within 72 h of collection.

A dataset with the original CellSearch analysis (including CTC and tdEV counts) and basic patient characteristics was provided by the Department of Medical Oncology, GZA Hospitals Sint-Augustinus. The CellSearch data were used for orthogonal validation of the AccuCyte-CyteFinder LEV identification method described herein.

For the present project slides prepared for the AccuCyte-CyteFinder analysis were re-analysed; both archived slides (stored at −80°C) and scanned images were used. All available samples without evidence of blood lysis and with available CellSearch and AccuCyte CTC data were included (N = 72). Baseline clinical characteristics of the included patients are summarised in Table 1.

**Table 1:** Patient characteristics of the metastatic breast cancer cohort.

Characteristic	Value
**Number of patients**	72
**Age, years**	Median 66 (range 33-98)
**Pathology**	
**Invasive ductal carcinoma (IDC)**	57
**Invasive lobular carcinoma (ILC)**	14
**NOS**	1
**Molecular subtype**	
**HER2-positive**	12
**ER-positive**	60
**PR-positive**	50
**Triple-negative**	8
**Sites of disease**	
**Local**	19
**Nodal**	21
**Lung**	18
**Liver**	15
**Bone**	51
**Brain or leptomeningeal metastases**	8
**Other**	21
**Number of metastatic sites**	
**1 site**	27
**2 sites**	30
**3 sites**	10
**4 sites**	3
**5 sites**	2

#### 2.1.3 Definition and identification of large extracellular vesicles with the AccuCyte-CyteFinder workflow

The archived images of fluorescently labelled blood smear slides (8 slides per patient) were re-analysed to identify and enumerate LEVs as defined above. Image galleries containing CK- and/or EpCAM-positive objects were reviewed using the CyteFinder II imaging platform. LEV identification was performed using predefined morphological and staining criteria derived from published descriptions of large extracellular vesicles. Although the MISEV guidelines define large extracellular vesicles (LEV) as nucleus-free, phospholipid-membrane enveloped structures that are larger than 200 nm in diameter [22], we applied a set of more stringent criteria to focus on a sub-population of LEVs that are specifically released by tumor cells, relying on the identification criteria of large oncosomes [5], [11]. LEV detection was limited to objects that are between 1 and 10 μm in diameter and positive for one or more tumor markers (such as CK/EpCAM). Although our operational definition of LEVs is consistent with that of the large oncosomes, tissue origin cannot be ascertained.

Structures consistent with large membrane-bound vesicle-like objects were annotated for further analysis. Image review and annotation were performed retrospectively by trained investigators blinded to CTC counts or survival outcome data. LEV annotation was performed by a single trained reviewer blinded to clinical outcome.

#### 2.1.4 LEV enumeration workflow optimization and sample analysis

An initial set of 12 patient samples, selected randomly from the cohort were analysed by enumerating all eight slides for each patient and record LEV counts for each slide separately. Concordance of total LEV counts with LEV counts extrapolated from sub-sets of slides was evaluated. Sub-sets including 6, 4, 3, or 2 slides were evaluated for each patient. Concordance criteria were Spearman's rho >0.95 between data sets, with standard error < 25% for each slide set. Bland-Altman analysis of log-transformed data was used to uncover systematic errors between enumeration methods.

The established criteria were validated on a set of 24 randomly selected patient samples and applied to the rest of the samples in the cohort. All LEVs on the slides were counted unless the count per slide was above 500. In those cases (N = 4), LEV count was capped at 500 per slide, the total count set to 4000.

#### 2.1.5 CellSearch tdEV analysis

Blood samples were analysed on the CellSearch platform. Tumour-derived extracellular vesicle (tdEV) counts were obtained through retrospective analysis using the ACCEPT algorithm [14]. tdEVs were defined as CK^+^/CD45^-^/DAPI^−^ objects with a diameter below 14 μm, and an eccentricity index of ≤0.8.

#### 2.1.6 Statistical analysis

Correlations between analytes were assessed using Spearman's rank correlation coefficient (ρ). Survival analyses were performed using Kaplan-Meier estimates and compared with the log-rank test. Differences between biomarker-based subgroups (HER2 and hormone receptor positive vs. negative subgroups) were evaluated using the Kruskal-Wallis test with Dunn's post hoc correction.

Univariable Cox regression models were first performed separately for LEV counts, CTC counts, and biomarker statuses to assess their individual associations with OS. Multivariable Cox regression model included LEV count, hormone receptor status (ER + or PR+) and HER2 status to evaluate their independent associations with overall survival. In a separate model, LEV count and triple negative status (TN) was evaluated.

Statistical analyses and graphs were performed using R (version 4.2.1).

## 3 Results

### 3.1 Development of large EV identification criteria on the RareCyte platform

Identification criteria for large EVs were established on the RareCyte platform following published definitions of large EVs [22], [23]. Specifically, candidate events were required to be CD45^−^, nucleus-free, positive for CK/EpCAM, and morphologically compatible with membrane-delimited objects.

**Fig. 1:**
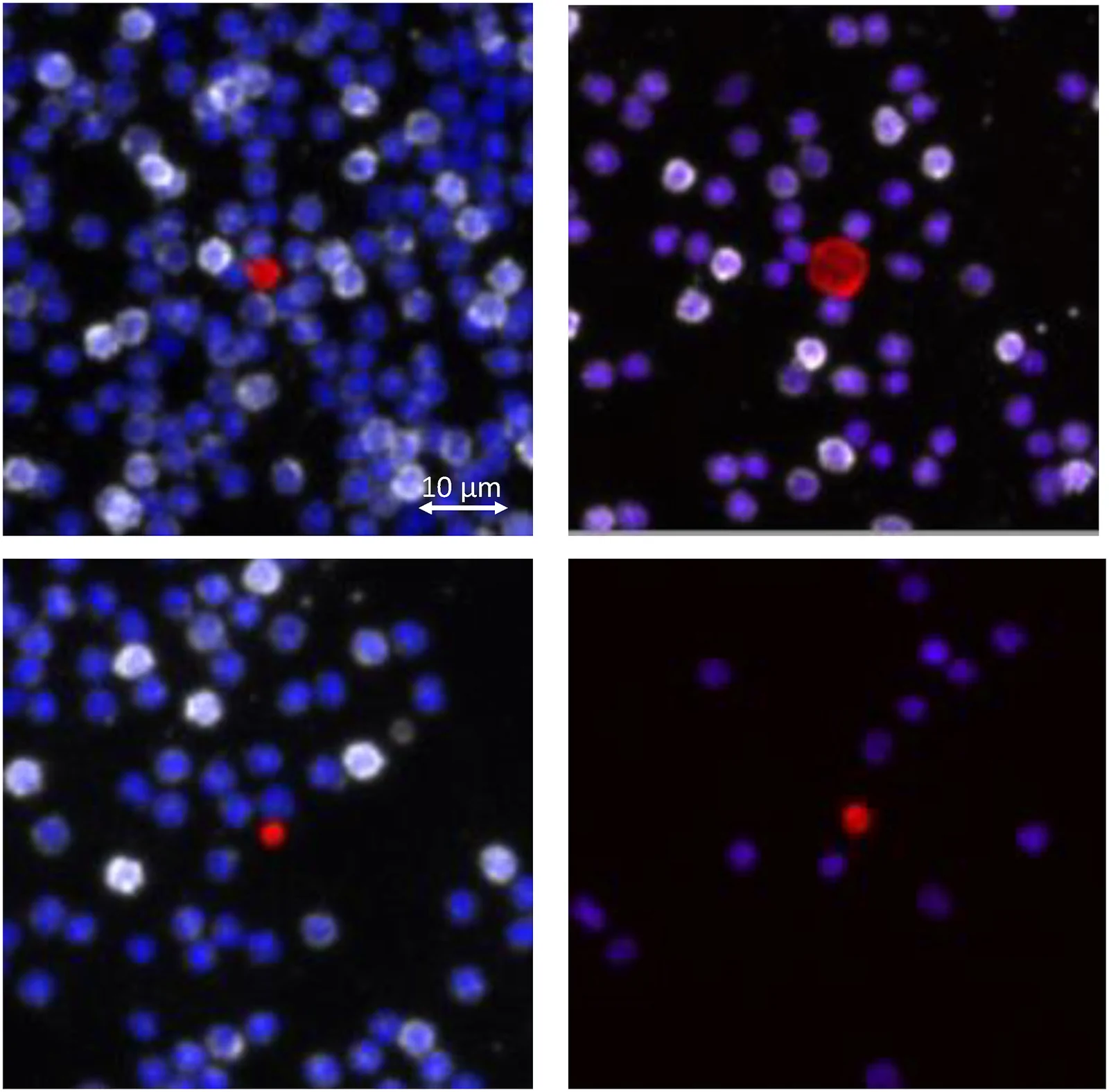
**Representative images of large EVs identified on the RareCyte platform.** LEVs were defined as CD45^−^, nucleus-free, CK- and/or EpCAM-positive vesicles ≥4 μm with smooth, sharp edges and round or oval shape. Blue: Nuclear stain, white: CD45 (WBC negative stain), red: CK/EpCAM. Scale bar = 10 μm. Representative objects from multiple MBC patient samples are shown.

To confirm the presence of a lipid bilayer membrane, 60 LEV-like objects identified across six patient samples were examined by brightfield microscopy at 40× magnification. The presence of a membrane could be reliably assessed in objects larger than 4 μm in diameter; this was established as the lower size cutoff for LEVs. Elongated objects (eccentricity >0.86) were also excluded. Additional criteria include a CK/EpCAM staining pattern to be consistent with a lipid bilayer-enveloped structure and the presence of a clear demarcation of the object's edge. Representative LEV images meeting these criteria are shown in Fig. 1.

As no molecular validation method was available for the analysed samples, the identified objects should be considered LEV-like structures based on combined morphological and immunofluorescence characteristics.

### 3.2 Optimization of the large EV enumeration workflow

As manual LEV enumeration from 8 slides per sample is labor intensive, we evaluated if accurate LEV quantification results could be obtained by extrapolating counts from a subset of slides. The pre-set criteria of Spearman's rho >0.95 for the complete vs. extrapolated sample sets and standard error below 25% for individual slide sets was achieved under the following criteria: three slides per patient enumerated unless the average LEV count per slide was equal or less than 7; in those cases, all slides were included in the analysis.

This workflow was validated on an independent set of 24 samples. Counts extrapolated from the analysis of three slides showed excellent concordance with manually enumerated 8-slide totals (Spearman ρ > 0.99; p < 0.001; Supplemental Fig. 1).

### 3.3 Validation of RareCyte LEV counts against CellSearch tdEV enumeration

**Fig. 2:**
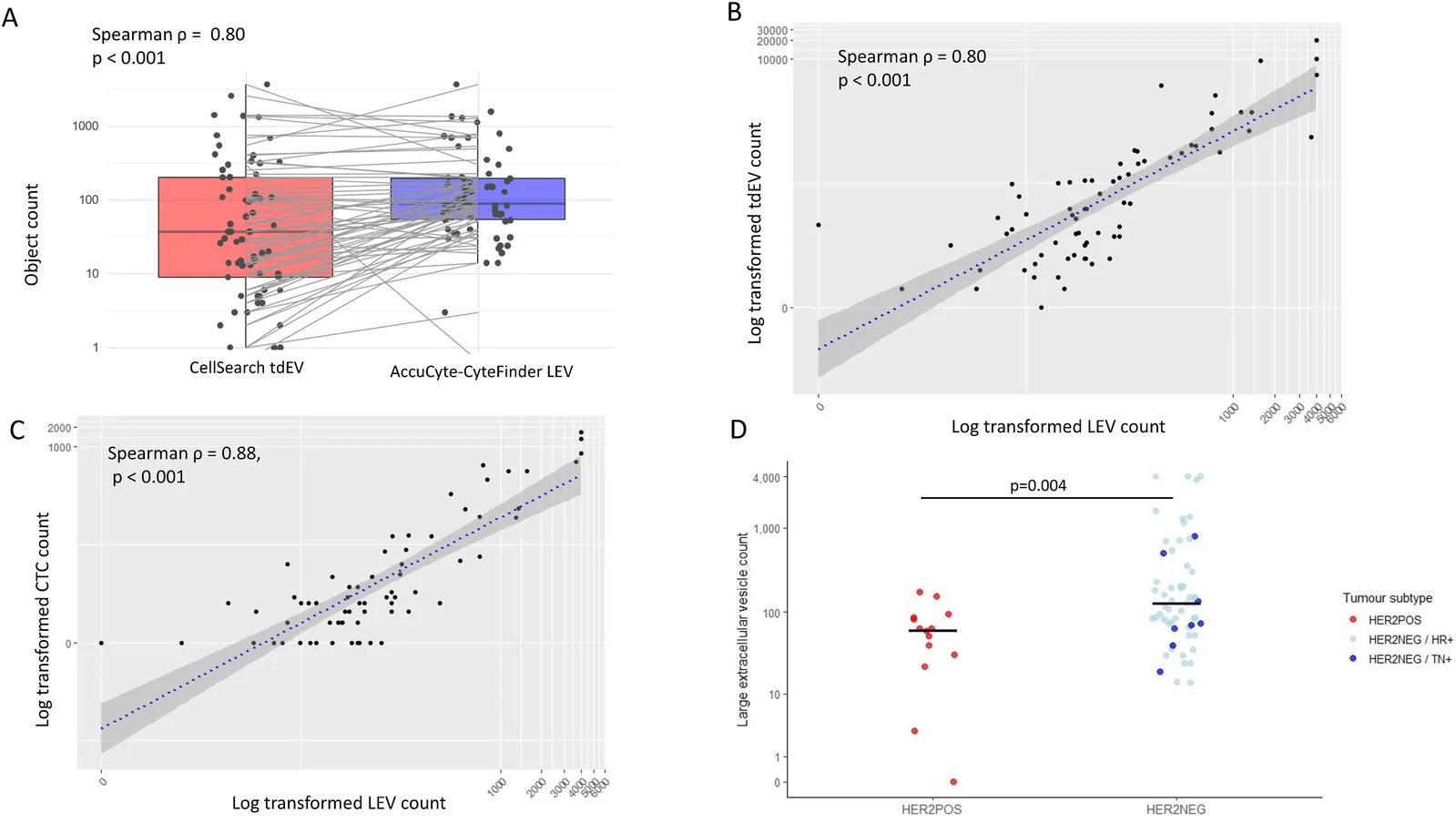
**Comparative characteristics of RareCyte large EV counts** A) Boxplot showing median LEV counts detected on the RareCyte platform and tdEV counts measured by CellSearch in 72 matched patient samples. B) Scatter plot showing positive correlation between LEV counts on RareCyte (X-axis) and tdEV counts on CellSearch (Y-axis). Both axes are displayed on a log1p-transformed scale to accommodate zero and low-range values. Individual data points represent samples, and the blue dashed line indicates the fitted linear regression model with its 95% confidence interval. (Spearman ρ = 0.80, p < 0.001). C) Scatter plot showing positive correlation between LEV counts and CTC counts measured on the same samples. Each point represents an individual patient. (Spearman ρ = 0.88, p < 0.001). D) LEV counts were significantly higher in patients that were categorized as HER2-negative based on their primary tumor (blue dots), than in those with HER2-positive tumors (red dots) (Kruskal-Wallis test, p = 0.004). Solid lines represent subgroup median LEV counts. Within the HER2-negative cohort, LEV counts did not differ significantly between hormone receptor-positive (HR+, depicted as light blue dots, N = 50) and triple-negative (TN) tumors (depicted as dark blue dots, N = 8) (Wilcoxon rank-sum test, p = 0.153).

Using the optimised workflow, LEV counts were obtained for 72 patients and compared with CellSearch tdEV counts from parallel blood samples. tdEV values were extracted from the original study dataset [21]. RareCyte LEV counts were significantly higher than CellSearch tdEV counts (median [IQR] 89 [53-208] and 37 [9-288], respectively (p = 0.007), Fig. 2A).

RareCyte LEV counts showed strong concordance with independently generated CellSearch tdEV counts from matched samples (Spearman's rho = 0.80; p < 0.001; Fig. 2B), supporting that both workflows identify a highly overlapping population of tumour-associated extracellular vesicle-like structures.

### 3.4 Correlation of LEV count with clinicopathological parameters in an MBC cohort

LEV counts using the RareCyte platform significantly positively correlated with CTC counts generated with the same platform (Spearman's rho = 0.88, p < 0.001, Fig. 2C), but were approximately 30-times more abundant; median [IQR] was 89 [53-208] for LEVs and 3 [0-21] for CTCs. In the CTC<5 subgroup (N = 41), median [IQR] LEV count was 69 [36-82], while median [IQR] CTC count was 1 [0-3]. In samples with no CTC (N = 22) detected, median [IQR] LEV count was 40 [20-88].

HER2 status showed that RareCyte LEV counts were significantly higher in patients with HER2-negative primary tumors (median [IQR] 127 [70-337] compared to patients with HER2-positive primary tumors (median [IQR] 89 [53-208]), p = 0.004, Fig. 2D), while CellSearch tdEV counts showed no significant difference. Further stratification of the HER2-negative group into hormone receptor positive (HR+) and triple negative (TN) subgroup revealed no significant difference between the median LEV counts of these subgroups (117 [68-337] vs. 69 [52-103] in HR+ and TN, respectively, p = 0.153).

The presence of distant metastasis was associated with a borderline statistically significant difference in LEV count (median 137 [IQR 77-498] vs. 89 [IQR 40-189], Wilcoxon rank-sum test, *p* = 0.05). The location of the metastases was not associated with differences in LEV count (Supplemental Table 2). No significant association was observed between histological subtype and LEV count (p = 0.120). (Supplemental Table 3). Associations with treatment modalities could not be evaluated due to substantial heterogeneity of treatment regimens, with 23 distinct treatment combinations among 72 patients.

### 3.5 Association Between large EVs and overall survival

In univariable Cox proportional hazards regression, increasing LEV count was associated with poorer overall survival (HR 1.04 per 100 LEVs, 95% CI 1.02–1.07, p = 0.0015). This association remained significant after adjustment for HER2, ER, and PR status (adjusted HR 1.05 per 100 LEVs, 95% CI 1.02–1.08, p = 0.0006), but lost predictive power when adjusted for TN status.

**Fig. 3:**
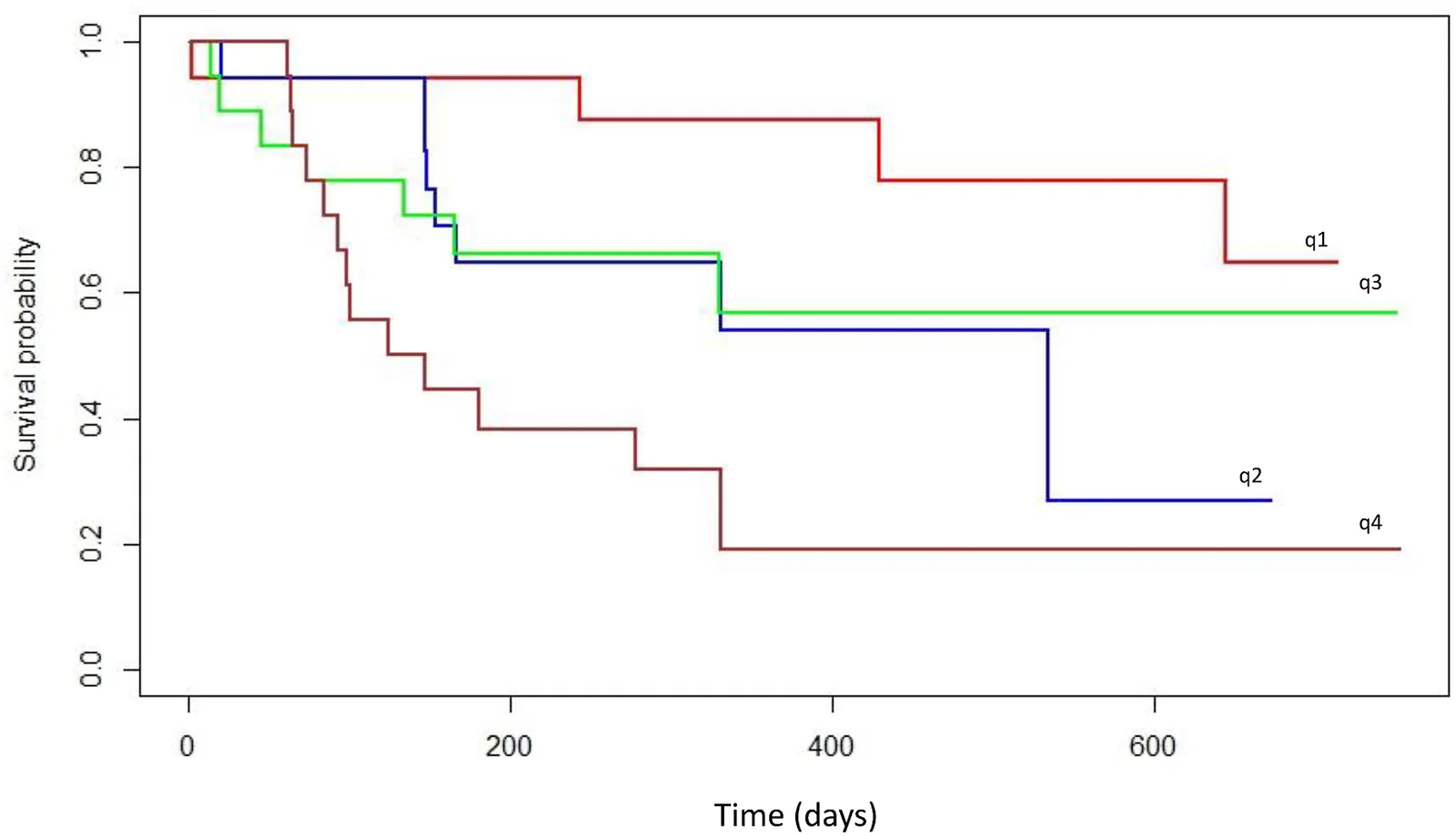
**Association of RareCyte LEV counts with circulating tumour cells and overall survival.** Kaplan-Meier curves for the metastatic breast cancer cohort (N = 72) stratified by LEV count into quartiles (red:Q1, blue: Q2, green: Q3, brown: Q4, N = 18 per quartile. (log-rank χ^2^ = 8.9, df = 3, p = 0.031).

Kaplan–Meier analysis using LEV quartiles demonstrated significantly different survival distributions (*p* = 0.030), with patients in the highest LEV quartile experiencing the poorest survival, those in the lowest quartile the most favorable survival, and those in quartiles 2 and 3 an intermediate survival Fig. 3.

Results of the univariate and multivariate Cox analyses can be found in Table 2.

**Table 2:** **Survival analyses of LEV count and clinicopathological variables** Summary of survival analyses evaluating the association between baseline large extracellular vesicle (LEV) count and overall survival in patients with metastatic breast cancer. Univariable and multivariable Cox proportional hazards regression models evaluated LEV count as a continuous variable, with hazard ratios (HRs) expressed per 100-LEV increase. The multivariable model included LEV count, human epidermal growth factor receptor 2 (HER2) status, estrogen receptor (ER) status, and progesterone receptor (PR) status. In a separate analysis, LEV count and triple negative (TN) status was evaluated. Kaplan-Meier analysis compared overall survival among patients stratified into LEV quartiles using the log-rank test. Hazard ratios with 95% confidence intervals (CIs) and corresponding *p*-values are shown for the Cox regression models. The Kaplan-Meier analysis reports the overall log-rank test comparing survival distributions across the four LEV quartiles.

Analysis	Variable	HR	95% CI	*p*-value
**Univariable Cox**	LEV count (per 100 LEVs)	1.04	1.02-1.07	**0.0015**
**Univariable Cox**	HER2 status	0.58	0.20-1.63	0.300
	ER status	0.34	0.16-0.72	**0.005**
	PR status	0.54	0.26-1.08	0.083
	TN status	2.86	2.44-6.62	**0.014**
**Multivariable Cox**	LEV count (per 100 LEVs)	1.05	1.02-1.08	**0.0006**
	HER2 status	0.84	0.28-2.50	0.753
	ER status	0.40	0.14-1.15	0.088
	PR status	0.66	0.27-1.61	0.363
**Mutivariable Cox**	LEV count (per 100 LEVs)	1.00	0.95-1.06	0.090
	TN status	2.87	1.23-6.68	**0.015**
**Kaplan-Meier (quartiles)**	LEV quartiles (Q1-Q4)	N/A	N/A	**0.030**^a^

a Log-rank test, χ^2^ = 8.90, df = 3.

We next explored whether LEV count could further stratify patients within established CTC prognostic groups. Patients were divided according to the accepted threshold of 5 CTCs per 7.5 mL of blood into CTC-low (<5 CTCs; n = 41) and CTC-high (≥5 CTCs; n = 31) subgroups. Median (IQR) LEV count was 69 [36–93] and 367 [127–1171], respectively. Within the CTC-low subgroup, higher LEV count showed a trend toward shorter overall survival (p = 0.061; Supplementary Fig. 2A). No significant association was observed within the CTC-high subgroup (p = 0.21; Supplementary Fig. 2B).

## 4 Discussion

Liquid biopsy technologies continue to evolve as complementary tools to tissue biopsy for disease monitoring and biomarker assessment in cancer, including metastatic breast cancer. Circulating tumour DNA (ctDNA) and circulating tumour cells (CTCs) are widely established liquid biopsy analytes, each with distinct advantages and limitations. ctDNA offers a stable, easily accessible source of tumour-derived genomic information but largely reflects DNA released from dying cells and its use is limited to mutation, copy number and methylation profiling. In contrast, CTCs represent the surviving tumour cells with metastatic potential and enable protein-level biomarker assessment, yet their low abundance often limits quantitative analyses.

In this study, we show that the CTC detection workflow of the RareCyte platform can reliably detect large EV-like structures (LEVs) in MBC patient samples at a nearly 30-times abundance compared to CTCs.

In concordance with previous studies [10], [15], LEV and CTC counts were highly correlated in our study, underlining the clinical relevance of LEVs identified with this method.

LEV counts obtained with this workflow were highly concordant with CellSearch tdEV counts from matched samples, supporting the notion that these analyses capture a highly overlapping population of tumour-derived vesicles. Differences in absolute counts between platforms reflect methodological differences; pre-analytical factors such as centrifugation parameters and staining conditions may affect vesicle recovery, but more importantly, the RareCyte method uses density-based, enrichment-free capture, while CellSearch relies on an EpCAM-based positive selection.

We demonstrated a significant, inverse relationship between LEV count and HER2 status of the primary tumor, in line with reports by others [3]. HER2-associated alterations in plasma membrane organization and tumor-cell phenotype may influence both the physical characteristics and immunophenotype of plasma membrane-derived extracellular vesicles. These changes could shift vesicles below the assay's size threshold or reduce CK/EpCAM expression, thereby decreasing the proportion of tumor-derived vesicles detected by our imaging-based workflow. Subgroup analysis of the HER2-negative group did not reveal statistically significant differences between hormone receptor positive and triple negative patients. As the triple negative cohort had a very limited size (N = 7), a larger study will be required to confirm how hormone receptor status impacts LEV count.

LEV counts generated using this workflow were also clinically relevant. Higher LEV counts were associated with shorter overall survival in the overall metastatic breast cancer cohort in both univariate and multivariate Cox regression analyses, and Kaplan–Meier analysis demonstrated significantly different survival distributions across LEV quartiles. These findings indicate that LEV quantification using the AccuCyte-CyteFinder workflow captures biologically meaningful information, consistent with previous studies demonstrating the prognostic value of CellSearch-derived tdEV counts in metastatic breast cancer and other tumor types [13,24]. In those studies, tdEV enumeration further refined risk stratification within both CTC-low and CTC-high patient groups. Although our exploratory subgroup analyses did not reach statistical significance, we observed a trend toward poorer outcomes among patients with fewer than five CTCs who had higher LEV counts, suggesting that LEV enumeration may also provide additional prognostic information beyond conventional CTC assessment. Larger studies will be required to validate the prognostic utility of LEV enumeration, determine clinically relevant cutoff values, and establish whether LEV counts provide incremental prognostic value beyond established CTC thresholds.

The potential clinical utility of LEVs may lie in biomarker monitoring applications. As they are directly derived from tumor cells and known to carry tumor markers [5], [7], analysing them can create novel insights into the tumor's biomarker status in the absence of tissue biopsy or analyzable CTCs. The RareCyte CTC workflow supports multiplexed fluorescent staining for the simultaneous detection of multiple biomarkers. The same workflows can be utilized to generate LEV biomarker data, offering a convenient complementary analyte to CTCs to detect clinically relevant biomarkers such as HER2 [25].

Notably, a large proportion of the patients included in this study (41/72, 57%) had fewer than five CTCs but a high number of LEVs, with a median of 69. In these patients analyzing LEVs can provide additional biomarker information that may not be obtainable through CTC analysis alone.

An important strength of this study is the use of an independent, previously validated CellSearch tdEV workflow as a parallel comparison method. The strong correlation between RareCyte LEV and CellSearch tdEV counts supports the validity of LEV detection across CTC imaging platforms.

Our study has several limitations. First, the analysis was retrospective and limited to a single metastatic breast cancer cohort. Second, LEV annotation relied on morphological and immunofluorescence-based criteria and was performed manually, which may introduce operator-dependent variability. Although strong concordance was observed with independently generated CellSearch tdEV counts, both workflows rely on related imaging- and immunofluorescence-based classification approaches. Orthogonal validation methods, such as structural or molecular characterization, would provide further confirmation of vesicle identity. Third, the lower size cutoff of 4 μm limits the analysis to a subset of circulating extracellular vesicles. Finally, platform-specific enrichment and staining differences between RareCyte and CellSearch may influence vesicle recovery and absolute counts.

In conclusion, we developed a workflow for the identification and quantification of large extracellular vesicle-like structures using the RareCyte CTC analysis platform. LEV counts correlated with both CellSearch tdEV and CTC counts and were associated with overall survival in metastatic breast cancer patients. Further studies are required to confirm the value of LEVs as complementary liquid biopsy analytes, particularly in patients with low CTC burden.

## Ethics declaration

Written informed consent to take part in the study and to publish the article has been obtained from all participants or their legal representatives. The privacy rights of participants have been observed.

This study included organ or tissue donors. This study includes human biological material and consent was obtained by donors, or their next of kin or legal representatives, for use in this study and for publication of the article. The samples used in this research were not sourced from executed prisoners or prisoners of conscience.

This study was performed in compliance with relevant laws, regulatory frameworks and guidelines where the research took place. This study was approved by the University of Antwerp. (Approval No. UAA11-18)

## Declaration of generative AI use

During the preparation of this work the authors used ChatGPT to improve text flow and brevity. After using this tool/service, the authors reviewed and edited the content as needed and take full responsibility for the content of the published article.

## Funding

This study received no external funding.

## Declaration of competing interest

The authors declare that they have no known competing financial interests or personal relationships that could have appeared to influence the work reported in this paper.

## Data Availability

The datasets generated and analysed during the current study are available from the corresponding author upon reasonable request. Access will be granted in accordance with institutional ethical regulations and data sharing agreements governing the original study [21].
