# Supplementary material for: A novel imaging-based approach for large extracellular vesicle detection and prognostic stratification in metastatic breast cancer
Source: J Liq Biopsy. 2026 Jul 30;13:100485. doi: 10.1016/j.jlb.2026.100485 (PMC13495395; doi:10.1016/j.jlb.2026.100485)
Supplement: Supplementary file 1 [file mmc1.docx]

**Figure S1. Concordance of total LEV count and extrapolated LEV count**

A) Scatterplot of total LEV count (enumerating all eight slides per patient) vs. extrapolated LEV count (enumerating three slides per patient unless the average LEV count is 7 or less). There is a strong linear correlation between the two sets of values (r=0.995, p<0.0001).
B) Bland-Altman analysis comparing extrapolated and total large extracellular vesicle (LEV) counts in the 24-patient validation cohort. The plot compares log-transformed total LEV counts (all eight slides) with log-transformed extrapolated LEV counts derived from the optimized slide-sampling strategy. The data demonstrated minimal systematic bias (mean difference = -0.01) with 95% limits of agreement of -0.25 to 0.22, corresponding to agreement within approximately **0.78 -1.24-fold** on the original count scale.

**Figure S2:** **Kaplan-Meier analysis in the subgroup of patients with favourable or unfavourable CTC counts.**
A) Kaplan-Meier analysis of patients with favourable (less than five) CTC counts (N=41), stratified by median LEV count (69) of this subgroup (red:“favourable”, blue: “unfavourable” groups). Patients with above-median LEV counts showing a trend (p=0.061) toward shorter overall survival in patients with higher LEV counts.
B) Kaplan-Meier analysis of patients with unfavourable (five or more) CTC counts (N=31), stratified by median LEV count (367) of this subgroup (red:“favourable”, blue: “unfavourable” groups). There was no significant correlation between LEV count and overall survival (p=0.21)

**Supplemental Table 1 -the impact of biomarker status on LEV counts**

Wilcoxon rank sum tests were performed to compare subgroups of the metastatic breast cancer cohort based on their HER2 and hormone receptor status

| Biomarker status | P value |
| --- | --- |
| HER2-positive | **0.004** |
| ER positive | 0.389 |
| PR positive | 0.325 |
| HR positive (ER and/or PR) | 0.3533 |
| Triple negative (TN) | 0.531 |

**Supplemental Table 2 - impact of site of metastasis on LEV count**

Wilcoxon rank sum tests were performed to compare subgroups of the metastatic breast cancer cohort based on the site of their metastases

| Metastatic site | *P* value |
| --- | --- |
| Local recurrence | 0.255 |
| Lymph node | 0.660 |
| Lung | 0.692 |
| Liver | 0.375 |
| Bone | 0.581 |
| Central nervous system | 0.936 |

**Supplemental Table 3 - impact of histology type on LEV count**

Kruskal-Wallis test was performed to compare subgroups of the metastatic breast cancer cohort based on the site of their metastases (χ² = 4.24, df = 2, p = 0.120)

| Histological subtype | n | Median LEVs (IQR) |
| --- | --- | --- |
| Invasive ductal carcinoma | 54 | 85 (60-170) |
| Invasive lobular carcinoma | 17 | 149 (53-696) |
| Mucinous ductal carcinoma | 1 | 4000 |
